# A Graph Based Framework to Model Virus Integration Sites

**DOI:** 10.1016/j.csbj.2015.10.006

**Published:** 2015-11-30

**Authors:** Raffaele Fronza, Alessandro Vasciaveo, Alfredo Benso, Manfred Schmidt

**Affiliations:** a Department of Translational Oncology, National Center for Tumor Diseases and German Cancer Research Center, Im Neuenheimer Feld 581, 69120 Heidelberg, Germany; b Department of Control and Computer Engineering, Politecnico di Torino, Corso Duca degli Abruzzi 24, 10129 Torino, Italy

**Keywords:** Gene therapy, Systems biology, Genomics, Insertional mutagenesis

## Abstract

With next generation sequencing thousands of virus and viral vector integration genome targets are now under investigation to uncover specific integration preferences and to define clusters of integration, termed common integration sites (CIS), that may allow to assess gene therapy safety or to detect disease related genomic features such as oncogenes.

Here, we addressed the challenge to: 1) define the notion of CIS on graph models, 2) demonstrate that the structure of CIS enters in the category of scale-free networks and 3) show that our network approach analyzes CIS dynamically in an integrated systems biology framework using the Retroviral Transposon Tagged Cancer Gene Database (RTCGD) as a testing dataset.

## 1 Introduction

Viral vector integration is a process exploited in gene therapy (GT) to correct defective cells of an individual and to drive the health status from the pathological condition to a normal one [1], [2], [3], [4], [5], [6]. As consequence of this perturbation, i.e. if vectors integrate into cellular genome positions where the expression of an important gene is dysregulated, the affected cell may step from the primary illness state to a secondary state. Thus, insertional mutagenesis is a potential risk that may accompany vector integration events [7], [8], [9], [10], [11].

Therefore, large insertional mutagenesis screenings are used to assess the safety of the treatment in clinical GT, to design safer GT protocols and to discover new disease (i.e. cancer) candidate genes [12], [13], [14], [15], [16].

The central role in integration site (IS) analyses is given in the assessment of the genome-wide integration profile and the identification of integration clusters that could alter gene expression. The definition of these clusters or common integration sites (CIS) is not standardized and usually based on accumulation of IS that are unlikely to occur by chance and statistically significant different compared to a random *in silico* control. A regular interpretation (Standard Windows Method, SWM) is founded on the number of integrations in a predefined genomic window, that classifies CIS as follows: a) 2 IS are within 30 Kb or b) 3 IS within 50 Kb or c) 4 IS within 100 Kb or d) ≥ 5 IS within 200 Kb [17], [18]. It is obvious that this historical definition can be used only as a first approximation for discovery of biologically (and clinically) relevant CIS, because the results are highly dependent from the size of the IS dataset [19]. Even if methods not constrained on a predefined set of fixed windows were released [20], [21], [22], [23], the data importation together with CIS generation and analysis remain tedious tasks. The static tabular and text oriented nature of the CIS representation requires extensive processing steps that can involve custom programming, format exchanging and manual interpretation of the results. These computational difficulties reduce the analysis capability of standard life science labs and strictly rely on elevated bioinformatics skills.

We hypothesized that in the next generation sequencing area only systems biology approaches may be able to dissect biologically (and clinically) relevant CIS. Here, we developed a new CIS construction framework using an approach based on graphs. This approach has numerous advantages: 1) the resulting CIS are represented by networks, 2) graph theory can be used to infer characteristics and properties of the integration process (i.e. the node degree distribution of the networks can be linked to the randomness of the integration process, and 3) a large repertoire of IS can be imported and parsed without any prior constraint (except for the maximal distance between two IS).

More in detail, the graph model allows an easy structural organization of the annotations in the Gene Atmosphere (GA) (e.g. protein coding atmosphere, post-transcriptional atmosphere, etc.) by using different layers of node categories while performing the enrichment as described in Paragraph 2.6. The implementation of this model in software tools, which allow networks visualization, provides a broader overview of the data at a glance. An example of this feature is depicted in Fig. 3 where the CIS network is disposed on a plane by applying a force-directed layout. Furthermore, with the availability of Hi-C data, this framework is ready to embed relations among CIS in the spatial organization of the genome, enriching the modeling to a multidimensional level [24] (i.e. moving from a linear genomic vicinity modeling to a topological genomic modeling). Another interesting feature which emerges using this graph model is the capability of assessing biological properties by exploiting topological characteristics of the network. For example, the scale-free distribution of a set of CIS can be used to establish if the dataset under analysis contains genomic regions enriched in IS, an observation that is a prerequisite in order to properly recognize CIS.

Recent approaches to the identification of hot-spots try to take into account the size of the IS's dataset and the prior knowledge about vector integration preferences [23]. The implementation of this graph model on a normal computer machine could be greedy of computational resources when dealing with very huge IS datasets (i.e. millions of nodes). To our knowledge, there are no IS datasets in literature big enough that cannot be easily represented by our model. Enhanced with annotated genomic data, this model could be easily extended in order to drive the identification of CIS exploiting the information contained in the annotations. It is our intention to evaluate this possibility in future. One of the main differences between our model and the statistical frameworks used to the identification of CIS is that here the statistical method is applied after the CIS identification leaving to the user of the model the ability to give a biological meaning to the CIS (e.g. the CIS is not excluded a priori by the statistical method). With the complex annotation feature, as described in Paragraph 2.6, our model is able to perform a many-to-many mapping against genomic features (i.e. genes) and integration sites while other methods just perform a one-to-one mapping (i.e. one gene, one IS). In this way, our model provides a more refined granularity, when it is enhanced with complex annotation, allowing the simultaneous representation of different genomic features in the model (i.e. transcriptional elements, protein coding genes, etc.), that other models do not allow.

A Cytoscape [25] draft prototype plugin was developed to test the framework of this paper.

## 2 Results and Discussion

### 2.1 CIS Definition

First of all we define what a common integration site is. A set of *n* IS in the database is represented as the set of *n* vertices *V* of the graph *G*. Then, for each couple of vertices *v*_*i*_ and *v*_*j*_ (*i*, *j* = 1, 2, …, *n i* ≠ *j*) we add an edge *e*_*ij*_ if the distance between the corresponding IS is below a threshold *T*_*H*_ of 50 Kbp. A weight *w*_*ij*_ is associated with the edge *e*_*ij*_ and represents the distance between the corresponding IS. The default value of 50 Kbp was selected using the maximal influence window size where a causal relation is found between an insertion event and gene expression [22]. De Jong showed that the presence of viral integration is correlated with the local amount of gene expression and that 50 Kbp is an upper bound on which the presence of IS can be linked with gene expression. At the end of this process, we obtain the undirected weighted graph *G* = (*V*, *e*) as abstract representation of all the distance relations in the IS dataset. The graph *G* is composed by a set of unconnected subgraphs (Connected Components, CC). Each CC is the natural graph representation of a CIS in which the order is represented by the number of vertices.

### 2.2 Integration Process and Node Degree Distribution

The non-random character of virus and viral vector integration suggests the existence of sub genomic regions that are preferentially targeted. As many complex biological systems where many components interact together, also the viral integration process derives from intricate functional interactions that involve viral and host proteins/DNA. The behaviors of complex systems are captured by a characteristic of the network that is called scale-free property [26], [27], [28]. This property depends on the distribution of the nodes degree. The node degree is the number of edges that connect a node with the neighbors. The degree distributions of several networks follow a power law, precisely defined with the functional *d*(*k*) = *ak*^− *γ*^, where *d*(*k*) is the degree distribution, *k*= 0,1,2,… is the node degree, *a* is the normalization constant and *γ* is the degree exponent. In scale-free network the exponent is usually less than three (*γ* < 3), whereas in random networks *γ* ≥ 3.

To prove that the mechanism of viral CIS or hot-spot (HS) formation is embedded via scale-free property into the network representation, we developed a series of synthetic transfection experiments that consisted of placing a fixed number of integrations on human genome carrying a random number of artificial hot-spots. The integrations were divided in two subsets: 1) IS placed on a simulated genome with hot-spots (IS_SYN_) and 2) IS randomly placed on a genome without hot-spots (IS_RAND_). The scale-free property of CIS networks found in IS_RAND_ and IS_SYN_ was then verified using the Cytoscape “Network Analysis” plugin.

**Fig. 1:**
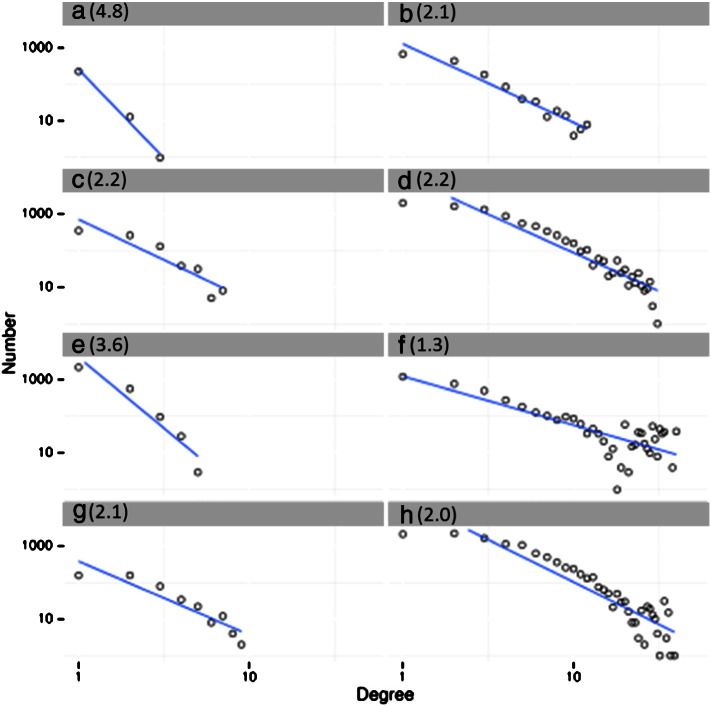
Degree distributions for datasets used to test scale free properties of CIS networks. The degree distribution of a scale-free network follows the power law *d*(*k*) = *ak*^− *γ*^, with *γ* < 3. This distribution is interpolated as a straight line on log–log plot.

We further verified the presence of a HS driven mechanism on six datasets: five in which we expected a scale free behavior (LV [1], HIV [29], GV1 [2], GV2 [16] and RTCGD [12]); and one from an adeno-associated viral (AAV) vector study [30] where we expected a random integration profile. In Fig. 1 the degree distributions of the groups of the experimental IS sets are plotted. The richness in integration sites of the datasets is: ~ 1000 IS_SYN_ (g), ~ 15,000 IS_RAND_ (e), ~ 4000 IS_LV1_ (b), ~ 2000 IS_AAV_ (a), ~ 35,000 IS_HIV_ (d), ~ 15,000 IS_GV1_ (h), ~ 800 IS_GV2_ (c), and ~ 8800 IS_RTCGD_ (f). All the experimental and synthetic sets, except for the AAV and RAND set, have a log–log degree distribution that follows a power law with gamma exponent γ < 3. Only two datasets, the random dataset IS_RAND_ (γ = 3.6) and IS_AAV_ (γ = 4.8) have no scale-free degree distribution. This last finding is in line with our and other published studies that did not attribute to AAV any HS driven integration pattern [30], [31]. From a practical point of view and as a first result of our graph modeling, the node degree distribution in a network that represent integration events indicate the presence of an accumulation process driven by genomic hotspots.

Réka [26] demonstrated that complex systems that display a high degree of error tolerance (robustness) are represented by scale-free networks. An incomplete IS dataset can be seen as the result of a process that remove IS from the complete basin of integrations present in a sample, due to unavoidable experimental subsampling. Recalling the robustness property for scale free networks we can prove that genomic hot-spots are identified even within an incomplete set of experimental IS.

### 2.3 General Structure of the CIS Pool and RTCGD Dataset

The Retroviral Transposon tagged Cancer Gene Database (RTCGD; http://variation.osu.edu/rtcgd/, [12]) was used as test case for our graph model.

**Fig. 2:**
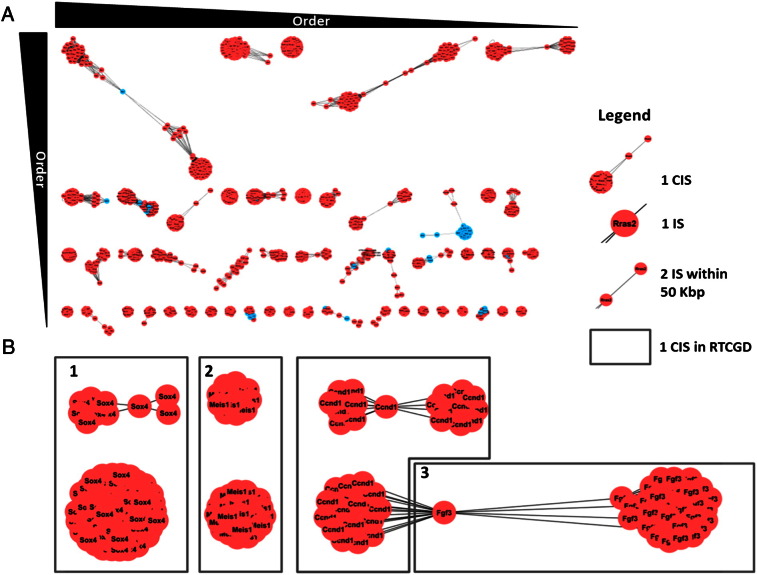
A) CIS representation on graphs of RTCGD. A CIS is represented as a network of nodes that symbolize a single integration event. An edge between two nodes indicates that the two corresponding IS are within a range of 50 Kbp. B) Comparison between three high order CIS in graph representation and original RTCGD.

The RTCGD dataset has been first analyzed in order to compare two general CIS properties, the order and the dimension of the 10 biggest CIS identified by our framework and the SWM. Fig. 2(A) shows the general structure and shape of all the CIS with order bigger than 9 as they appear analyzing RTCGD integration.

5110 IS are selected by the CIS construction tool as belonging to reputed CIS and 4035 compose CIS with p-value < 0.05 (see Appendix A Table 1 in [41]). How the p-value is computed per CIS is explained in the Paragraph 3.6. The CIS order goes from 2 to 82 (in Fig. 2(A) CIS from order 2 to order 8 are not shown). RTCGD data contains 2910 IS and the CIS falls in the same range order. No statistical model is applied in order to test the CIS significance.

In the 10 biggest CIS the order and dimension of 3 of them (*myc*, *ahi* and *rasgrp1*) were returned identical by the analysis performed using our model and RTCGD and other 4 CIS (*gif1*, *lvis1*, *pim1* and *notch1*) were comparable (difference in the order is less than 10; see Table 1). For the remaining common integration sites the RTCGD ones were fragmented in two separated CIS by using our model (*sox4* and *meis1*) or vice versa (*fgf3*). Fig. 2(B) shows the structure of these CIS. A box surrounding two CIS indicates that the CIS are combined in RTCGD.

The CIS discovered using our graph model have a wide spectrum of dimensions imposing only the threshold distance between two IS. As shown in Table 1 the high order CIS are generally more compact than the CIS retrieved by a SWM. This behavior can easily explained by the construction rules that SWM imposes to fill a region of 200 Kbp in CIS with order bigger than four.

**Fig. 3:**
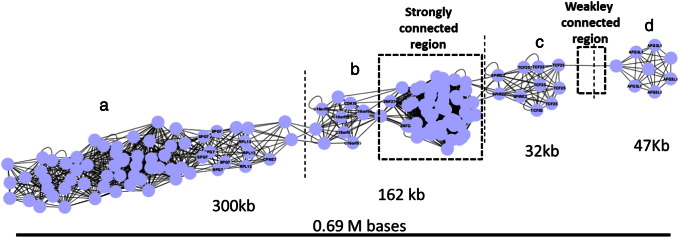
The network representation of the biggest CIS (0.69 Mbases) in IS_HIV_ (chromosome 16: 89,730,888; ~ 35,000 IS). The structure contains some regions with many (a, b, c and d) and few (e) integration sites. Labels in the nodes correspond to genes in the original IS annotation.

**Fig. 4:**
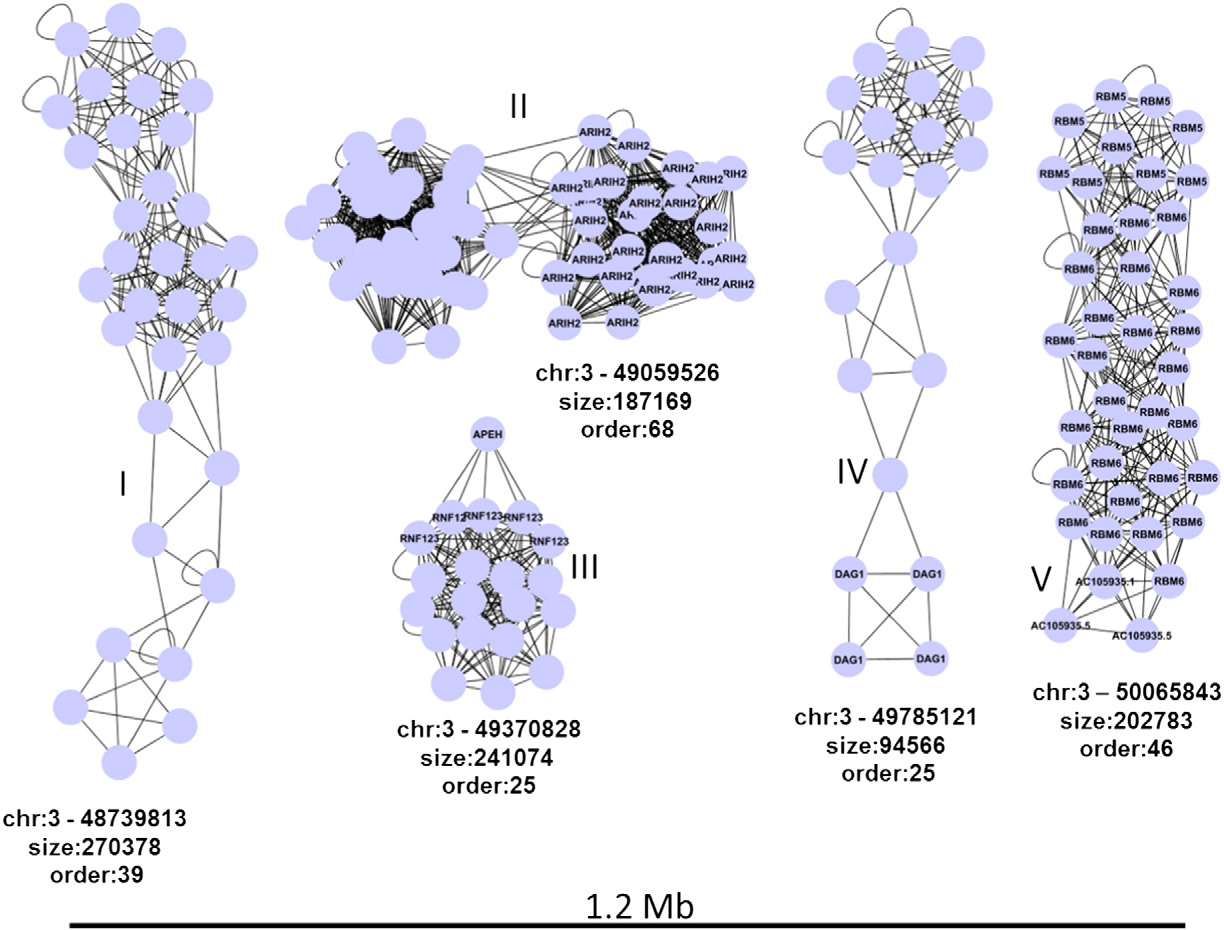
5 independent CIS of order 25–68 are detected in a IS dense region of 1.2 Mbases (chromosome 3:49,000,000–50,000,000). Three of them (I, II and IV) are composed by at least two sub regions. Labels in the nodes correspond to genes in the original IS annotation.

As a further point of discussion we would like to make some observation on the structure of CIS in sets of IS that contains many IS that accumulate in genomic hotspots that cover several megabases. The IS_HIV_ set of IS contains more than 35,000 objects and produces a typical integration profile with dense accumulation regions. The biggest CIS is found on chromosome 16 at position 89,730,888 and spans 0.69 Mb. The graph that represents this hotspot is shown in Fig. 3 and is strongly anisotropic. In order to arrange the nodes in this manner we used a force-direct layout embedded in Cytoscape. Accumulations of nodes (strongly connected) indicate an area in the CIS rich in integration. Regions in the network where few links (weakly connected, 3e) are present represent areas that act as interface between two IS dense areas. The CIS in the figure might be dissected in at least 4 independent subregions that cover 300 (3a), 162 (3b), 32 (3c) and 47 (3d) kilobases and where different genomic elements are targeted. In Fig. 4 the networks structure of a region of 1.2 Mb on chromosome 3 is shown. Five independent CIS of order 25–68 are detected. Only one of them 4II is isotropic and does not show subregions of accumulation. Three CIS, 4I, 4II and 4IV, contain at least two subregions whereas in 4V, 46 IS cover more or less uniformly a region of about 200 Kb. To conclude this paragraph, the networks can be visually represented to be easily examined by eye or is possible to use graph algorithms that automatically extract topological structures related with specific hotspot features.

### 2.4 Shannon Index and Dataset Comparison

Entropy [32] is the most elementary concept of information theory and is widely used in many fields in order to measure the complexity of a defined system. We can introduce a level of complexity to the CIS analysis combining the IS that belong to different investigational classes like tumors or vectors type. A hotspot can consequently be composed by a mixture of IS that derive from different sources. In order to measure the complexity of one CIS we computed the entropy value, absolute and relative. Low entropy values are found in CIS where IS come from few tumor source (exactly one source if normalized entropy *NE* = 0). If *NE = 1* all the tumor sources contribute equally to the CIS. To determine significant tumor hotspots we extracted the set of CIS that belong to 10% of the upper (highly heterogeneous) and lower (highly homogeneous) tail of the entropy distribution. The *NE* distribution for 203 CIS (order > 4; 2129 IS) is comprised between 0 and 0.65 (median = 0.4). 23 CIS (201 IS, 9.4%) with *NE* < 0.1515 and 18 CIS (198 IS, 9.3%) with *NE* > 0.5375 were selected as homo and heterogeneous CIS respectively. The largest homogeneous CIS was found on chromosome 15: 98795118 (Wnt1 gene) and contains 28/29 IS that are associated with mammary tumor. The most heterogeneous CIS was found at the position chr2:173665 (*cebpb* gene) and contains 23 IS. It is originated from 6 different tumors: T cell, B cell, myeloid, lymphoma, brain tumor and HS. On average the CIS order and dimension were 11 and 99.5 Kbp respectively in the heterogeneous set and 9 and 58 Kbp in homogeneous CIS.

### 2.5 Literature Analysis

A detailed literature exploration of the list of genes returned in the homogeneous set was performed in order to detect potential candidates to further experimental tasks. The probability that a homogeneous CIS appears by chance was assessed on a multinomial model. The complete list of homogeneous CIS is given in Table 2. We divided the genes present in this list in 3 collections concordantly with the CIS homogeneity: 1) in the “mammary” group those found in “mammary tumor”, 2) in the “sarcoma” group those found in “sarcoma” and 3) combining in the “Leukemia” collection those found in “B cell”, “T cell” or “myeloid”. The analysis was performed using ProteinQuest, a literature mining tool [33], [34].

All the genes found specifically for mammary tumor were already well known as contributing to mammary neoplasia. In the sarcoma group genes, *tmem74* and *rabgap1l*, only *rabgap1l* was previously linked with tumors [35], but not with sarcomas, while in the leukemic group *usp49*, *smsg6* and *zpf423* were not linked with blood diseases.

### 2.6 Complex Annotation

The previous investigation is directly based on the gene annotation provided by RTCGD, annotation found using the Next Gene Approach (NGA). This approach just annotates an IS with the name of the proximal genetic element, that habitually is a protein coding genes. This procedure implicitly force a many-to-one relation between IS and a gene element. We think that this is a true analysis limitation because it is not possible to assign multiple genes to the same IS. Moreover, due to the variety of the gene types (i.e. protein coding, miRNA or lincRNA), to the complexity of genetic loci (i.e. introns can contain non coding genes) or simply to the high density of gene elements in few kilo bases, the NGA should be limited only to a raw estimation of the genes involved in the viral integration. In our network model we decided to introduce a new category of nodes that represent gene elements. These auxiliary nodes added to the network symbolize genes found within a certain distance (50 Kbp by default) to any IS. With this simple addition to the network structure we naturally introduced a many-to-many relation between integration sites and genomic elements (referred as Transcriptional Elements (TE) in the model). The group of the genes found in one CIS network is defined as the gene atmosphere (GA) of the CIS. Applying this new network layer, we were able to notice many other gene elements that did not emerge in the previous literature analysis.

In the following analysis we focus only on genes found in GA (see Appendix A Table 2 in [41]) that are not present in the original RTCGD annotation.

#### 2.6.1 Sarcomas

**Fig. 5:**
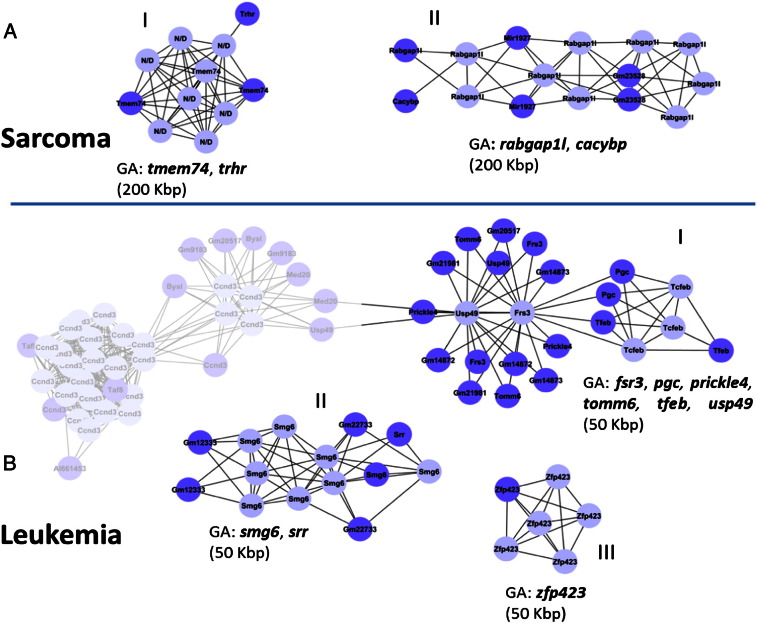
Gene atmosphere of the homogeneous CIS associated only with A) Sarcomas or B) Leukemia but not linked with the same disease in the literature analysis. Purple nodes: genes, light blue: insertion sites.

No gene elements were found in sarcoma CIS Fig. 5. AI and AII using 50 Kbp threshold. For this reason we increased the threshold to 200 Kbp. Two coding genes, *tmem74* and *trhr*, constitute the GA of this CIS. The first was reported in RTCGD and used to annotate the CIS. The other one not reported in the original list, the thyrotropin-releasing hormone receptor gene (*trhr*), was associated with differentiated thyroid cancer risk [36]. In CIS AII the composition of the GA contains two coding genes (*rabgap1l*, *cacybp*), 1 miRNA (mir1927) and 1 snoRNA (GM23528). The calcyclin-binding protein (*cacybp*) was ubiquitously detected in all kinds of tumor tissues and was highly expressed in nasopharyngeal carcinoma, osteogenic sarcoma, and pancreatic cancer [37].

#### 2.6.2 Leukemia

As displayed in Fig. 5.BI two CIS fall in the same locus. The two twin CIS are falsely connected for the reason that IS belonging to distinct CIS are coupled over two common genes (*med20* and *usp49*). The GA of BI contains 6 coding genes *fsr3*, *pgc*, *prickle4*, *tomm6*, *tfeb*, *usp49*. Interestingly this block of mouse genes was found and described in a human large linkage disequilibrium block, which contains CCND3, BYSL, TRFP, USP49, C6ofr49, FRS3, and PGC [38]. This block contains somatic alterations that correlate with breast cancer prognosis and survival in humans. In CIS BII the GA contains *smg6* and *srr*. We didn't find any verified involvement of *srr* in tumor development. In CIS BIII we found complete concordance between our graph model complex annotation and RTCGD because only *zfp423* compose the GA.

Therefore, implementing an annotation strategy based on a single simple network construction rule, we were able to explore several potential tumor related genes both coding and not coding that are not described in the original dataset.

We would like to briefly illustrate a pathway analysis that we performed on 1421 unique genes found in the GA (see Appendix A Table 2 in [41]). Processes associated with the chromatin rearrangement and the ribosome structure emerge clearly from the data. Because all the IS in RTCGD are strongly correlated with the appearance of tumors in mice, the likelihood that genes in the neighborhood of an IS are in a significant pathway is in fact high. Consequently, we do not discuss further this result.

In conclusion in this work was our interest to show that the network representation of integration sites hotspots is a convincing framework since it links topological structures of the graphs with biological features of the CIS. To demonstrate that the complex interactions between viral and cellular components during the viral integration originates the IS accumulation, we showed that the graphs representing putative hot-spots are represented as scale-free networks. We propose that the visual modeling introduces a better data understanding and that the incorporation of the model in frameworks that manage graph representation of biological data, like Cytoscape, makes easier to explore data and test alternative functional hypothesis.

## 3 Experimental Procedures

### 3.1 Integration Sites Datasets

Six datasets, LV, HIV, GV1, GV2, AAV and RTCGD were obtained from the authors of the studies [1], [2], [12], [16], [29], [30] whereas two random datasets were created as described below.

### 3.2 Liftover of RTCGD from mm9 to mm10

RTCGD dataset (http://variation.osu.edu/rtcgd/) is actually based on mm9 genome assembly. The dataset was updated to mm10 assembly via liftover procedure [39]. A conservative minimum ratio of bases remapping was chosen (0.95) along with unique output regions in order to guarantee the quality of the mapping. From 8807 IS only two were not mapped on the new genome assembly.

The gene annotation table based on Ensembl GRCm38.p2 was used for complex annotation

### 3.3 Network Construction

A graph is constructed following these steps:

CIS Layer:
  1. Order the Insertion Site Dataset*D*by Insertion Position (location)
  2. For each IS*i*in the ordered Insertion Site Dataset*D*do
    2.1. Add a node*v*_*i*_, annotating the location of the IS*i*to the graph*G*_*D*_
    2.2. For each node*v*_*j*_in the graph*G*_*D*_do
      2.2.1. If the location of*v*_*i*_is at a distance smaller than the threshold*T*_*H*_from the node*v*_*j*_,with*i* ≠ *j*
        a) Connect the*v*_*i*_and*v*_*j*_with the edge*e*_*ij*_and weight*w*_*ij*_, add*e*_*ij*_to graph*G*_*D*_
        b) Otherwise continue from 2.2 (next *j*)
    2.3. Continue from 2 (next *i*)
GA Layer:
  1. Order the Transcriptional Element Dataset*D*_*TE*_by Transcription Start Site (location)
  2. For each TE *i* in the ordered Transcriptional Element Dataset*D*_*TE*_do
    2.1. Add a node *v*_*i*_, annotating the location of the TE*i*to the graph *G*_*D*_ (previously created and ordered by location)
    2.2. For each node *v*_*j*_in the graph*G*_*D*_do
      2.2.1. If the location of*v*_*i*_is at a distance smaller than the threshold *T*_*H*_from the node*v*_*j*_, with*i* ≠ *j*
        a) Connect the*v*_*i*_and*v*_*j*_with the edge*e*_*ij*_and weight *w*_*ij*_, add*e*_*ij*_ to graph*G*_*D*_
        b) Otherwise continue from 2.2 (next *j*)
    2.3. Continue from 2 (next *i*)

Scale free structure of graphs is tested using NetworkAnalyzer plugin [40] directly in Cytoscape.

### 3.4 Random Datasets and Random CIS Statistics

In order to detect integration patterns that deviate from a random even distribution we generated a set of *m* = 100 datasets composed of *l* = 500, 2000, 5000, 10, 000 random IS. Two factors were taken into account in order to avoid some biases in the random model: 1) only regions reported as mappable by 100 bp reads (http://moma.ki.au.dk/cgi-bin/hgTables) are chosen as potential IS areas and 2) the fraction of IS per chromosomes is shaped on the frequency detected in the LV1 studio. From the created random datasets we extracted (connection threshold 50 Kbp) and grouped the CIS by the order *i* = 2,.., 10. In the random dataset we never detected CIS with order larger than 9. On these groups we computed 1) the CIS rarity distributions (see Paragraph 3.6), and 2) the maximal number of random CIS of order *i* found in any of the *m* the datasets of numerosity *l*. Except for random CIS of order *i* = 2, where *R*_*l*2_ uniformly distributed over the threshold distance, the distribution of *R* used to compute the p-value and log-likelihood was approximated by a continuous normal distributions of mean *µ*_*θrnd*_ (26,000 bases) and standard deviation *σ*_*θrnd*_ (12,000 bases). These values were chosen from the smallest mean and biggest variance found within all the *R*_*li*_. Also for the experimental CIS we approximated the rarity distribution using mean and variance found in LV1 samples.

### 3.5 Synthetic Transfection Experiment

An in silico chromosome that contains a fixed number of randomly placed hotspots was created. In brief, 159 hot-spots with various dimensions (from few hundred bp to 5 Mbp) where randomly marked on a linear chromosome composed by 100 Mbp. A set of 100, 500 and 1000 insertion sites were simulated on this chromosome. A random process then assigns a fraction of IS randomly in the hotspots (H_is_) and a fraction on the complete chromosome (H_rnd_). We decided to use a ratio H_is_: H_rnd_ = 1:3 between random IS and hotspot IS in order to simulate an experiment with a low signal to noise ratio.

### 3.6 Statistical Model, p-Value and Log-Likelihood Ratio Test

For each CIS a p-value and a log likelihood ratio for a 2-class problem, with full specified null (*θ*_*rnd*_) and alternative (*θ*_*ex*_) hypotheses, are returned.

Two classes of CIS are extracted from the data: CIS_RAND_, the CIS found in the simulation experiment without hot-spots (IS_RAND_) and CIS_EXP_, the experimental CIS found in one clinical study [1]. The simulation experiment was repeated 100 times (connection threshold 50 Kbp) in order to obtain a measurement of the CIS frequency.

The rarity *R* measures the IS compactness in a CIS and the rarity distributions were computed for both CIS classes. The rarity for dataset *l* and order *i R*_*li*_ is expressed as $\frac{\mathrm{dimension}\;\mathrm{of}\;\mathrm{the}\;CIS\;\mathrm{in}\;\mathrm{datatset}\;l}{i-1}$. Except in random CIS of order 2, where *R* uniformly distributed over the threshold distance, the rarity was approximated by two continuous normal distributions of mean (*µ*_*θrnd*_, *μ*_*θex*_) and variance (*σ*_*θrnd*_^2^, *σ*_*θex*_^2^).

Two prior probabilities for the hypothesis, *p*(*θ*_*ex*_) and *p*(*θ*_*rnd*_), were calculated from the number of experimental (*N*_*ex*_) and expected (*N*_*rnd*_) CIS. The expected number of random CIS, grouped by order, was equated to the maximal CIS frequency found in the random experiments for CIS of same order. Then the priori for the experimental class is *p*(*θ*_*ex*_) = *N*_*ex*_/(*N*_*ex*_ + *N*_*rnd*_) and *p*(*θ*_*rnd*_) = 1 − *p*(*θ*_*ex*_).

The probability of an error, interpreting a CIS as not random (rejection of *θ*_*rnd*_) given the CIS rarity *x*, is

$$p\left(\theta_{rnd}|x\right)=p\left(x|\theta_{rnd}\right)p\left(\theta_{rnd}\right)=p\left(\theta_{rnd}\right){\left(2\pi \sigma_{\theta_{rnd}}^{2}\right)}^{-\frac{1}{2}}\int_{-\infty }^{x}\exp\left(-0.5{\left(\frac{x-\mu_{\theta_{rnd}}}{\sigma_{\theta_{rnd}}}\right)}^{2}\right)dx\text{.}$$

We returned also the log-likelihood ratio Λ(*x*) in order to indicate the CIS class more probably given the rarity *x*.

For a 2-class problem the class *θ*_*ex*_ is chosen when *p*(*θ*_*ex*_|*x*) > *p*(*θ*_*rnd*_|*x*). By the Bayes theorem it is equivalent to write *p*(*x*|*θ*_*ex*_)*p*(*θ*_*ex*_) > *p*(*x*|*θ*_*rnd*_)*p*(*θ*_*rnd*_). Rearranging this relation we can write

$$\Lambda \left(x\right)=\log_{2}\frac{p\left(x|\theta_{\mathrm{ex}}\right)p\left(\theta_{\mathrm{ex}}\right)}{p\left(x|\theta_{rnd}\right)p\left(\theta_{rnd}\right)}$$

obtaining the decision rule that if Λ(*x*) > 0 the hypothesis *θ*_*rnd*_ is rejected. The higher is Λ(*x*), the better the is hypothesis *θ*_*ex*_.

This probability *p*(*θ*_*rnd*_|*x*) and the log-likelihood Λ(*x*) score are associated to any CIS and returned to the user.

### 3.7 Integration Sites Analysis Software and Availability of Supporting Data

Cytoscape 2.8 was used as prototyping environment to perform all the network examination. Supporting material as the prototype plugin for Cytoscape 2.8 and tutorials are available on request.

**Abbreviations**
CIS: Common Insertion Sites
GA: Gene Atmosphere
GT: Gene Therapy
HS: Hot Spot
IS: Insertion Sites
NGA: Next Gene Annotation
SWM: Standard Window Method

## Competing Interests

The authors declare that there is no conflict of interest.

## Figures and Tables

**Table 1:** RTCGD CIS order and dimension comparison. Only the 10 biggest CIS are considered. In parenthesis the order or the dimension of the CIS that complete the corresponding CIS found applying the dual method.

	Graph CIS		RTCGD	
CIS name	Order	Dimension	Order	Dimension
Gif1	81	122,521	82	197,286
Myc	78	234,094	78	234,094
Sox4	65 (10)	51,755 (130,119)	75	235,775
Ahi	61	297,769	61	297,769
Pim1	35 (9)	12,131 (81,543)	43	144,748
Meis1	26 (13)	9,894 (46,027)	38	154,229
Lvis1	42	118,556	38	89,966
Fgf3	53	143,856	38 (33)	95,144 (182,060)
Rasgrp1	36	86,755	36	86,755
Notch1	36	92,014	34 (2)	90,385 (1,629)

**Table 2:** Genes found in homogeneous CIS (normalized entropy, NE < 0.1515).

Chr	Position	Dimension	Order	Tumor type	Gene	Entropy	p-Value
15	98795118	65490	29	Mammary_tumor	Wnt1	0.055	< 0.001
3	97979620	120117	20	Bcell	Notch2	0.146	< 0.001
11	103817471	61740	12	Mammary_tumor	Wnt3	0.000	< 0.001
14	115040166	9438	12	Tcell	Mirn17	0.106	< 0.001
11	59292730	16451	10	Mammary_tumor	Wnt3a	0.000	< 0.001
1	160495222	156990	9	Sarcoma	Rabgap1l	0.129	< 0.001
4	3916043	43556	9	Myeloid	Plag1	0.129	< 0.001
7	25114306	15181	9	Bcell	Pou2f2	0.000	< 0.001
4	154549687	57802	8	Bcell	Prdm16	0.139	0.002
11	74982305	88850	8	Bcell	Smg6	0.000	< 0.001
13	37813516	74223	7	Bcell	Rreb1	0.139	0.002
15	43989356	57412	8	Sarcoma	Tmem74	0.000	< 0.001
12	86876152	126384	7	Bcell	6430527G18Rik	0.151	0.004
20	12087680	59992	7	Bcell	Bcor	0.151	0.004
16	30038826	74290	6	Bcell	Hes1	0.000	0.001
17	45556480	2280	6	Bcell	Nfkbie	0.000	0.001
2	31079892	40607	5	Bcell	Fnbp1	0.000	0.003
6	125321059	37420	5	Bcell	Ltbr	0.000	0.003
8	87959778	25262	5	Bcell	Zfp423	0.000	0.003
15	80537828	36086	5	Tcell	Grap2	0.000	< 0.001
16	24166711	82290	5	Bcell	Bcl6	0.000	0.003
16	39882896	180	5	Mammary_tumor	ENSMUSG00000068293	0.000	< 0.001
17	47754686	85300	5	Bcell	Usp49	0.000	0.003
